# Contemporary esophageal physiological testing for primary esophageal motility disorder (PEMD) and gastroesophageal reflux disease (GERD) before bariatric surgery: A systematic literature review

**DOI:** 10.1111/obr.13924

**Published:** 2025-03-26

**Authors:** Muhammed Ashraf Memon, Khorshed Alam, Zahirul Hoque, Shahjahan Khan

**Affiliations:** ^1^ School of Mathematics, Physics and Computing and Centre for Health Research University of Southern Queensland Toowoomba Queensland Australia; ^2^ Sunnybank Obesity Centre South and East Queensland Surgery (SEQS) Sunnybank Queensland Australia; ^3^ Mayne Medical School, School of Medicine University of Queensland Brisbane Queensland Australia; ^4^ Faculty of Health Sciences and Medicine Bond University Gold Coast Queensland Australia; ^5^ Faculty of Health and Social Science Bolton University Bolton Lancashire UK; ^6^ School of Business, and Centre for Health Research University of Southern Queensland Toowoomba QLD Australia; ^7^ School of Mathematics, Physics and Computing University of Southern Queensland Toowoomba Queensland Australia; ^8^ School of Science and Engineering Asian University of Bangladesh Dhaka Bangladesh

**Keywords:** ambulatory pH monitoring, bariatric surgery, esophageal dysmotility, gastroesophageal reflux disease, high resolution impedance manometry, morbid obesity

## Abstract

**Objective:**

This systematic review was conducted to evaluate the preoperative prevalence of primary esophageal motility disorders and gastroesophageal reflux disease in patients with morbid obesity before bariatric surgery.

**Background:**

The use of esophageal manometry ± 24‐hour pH study before bariatric surgery was explored.

**Material and Methods:**

Articles on preoperative conventional or high‐resolution manometry ± 24‐hours pH‐study or both before bariatric surgery between 1999 and 2023 were identified using the Medline, PubMed, EMBASE, Cochrane Register of Systematic Reviews, and Science Citation Index. The search terms were selected for each search engine to optimize the published literature and meet the inclusion criteria. The modified AXIS was used as a critical appraisal tool to assess the quality of studies.

**Results:**

Thirty‐three studies performing preoperative esophageal manometry ± pH studies or both were identified. Various manometric abnormalities have been described by the authors depending on the type of technique used. Twenty‐two studies undertook a 24‐hour ambulatory pH study to identify abnormal acid exposure. Twenty studies performed preoperative gastroscopy. The incidence of hiatal hernia varied from 5.4% to 52.6%, and reflux esophagitis from 4.4% to 42%.

**Conclusions:**

The preoperative prevalence of PEMD and GERD was significant in patients with morbid obesity. This implies that the selection of the most appropriate bariatric procedure needs to be tailored not only for weight reduction but also for the prevention of further deterioration in esophageal motor function and GERD and its future consequences, such as Barrett's esophagus, erosive esophagitis, and esophageal adenocarcinoma, in both the short and long term.

## 1 INTRODUCTION

The incidence of primary esophageal motility/motor disorder (PEMD) using esophageal manometry, a physiological diagnostic test that evaluates the motor function and pressure of various parts of the esophagus, is as high as 61% in individuals with morbid obesity compared to individuals who are not obese.^1^ There are several different types of PEMD observed in persons with morbid obesity depending on the type of manometry (i.e. conventional or high resolution) and version of the Chicago Classification utilized. These include esophagogastric junction outflow obstruction (EGJOO), jackhammer esophagus (JE), nutcracker esophagus (NE), diffuse/distal esophageal spasm (DES), ineffective esophageal motility (IEM), and hypotensive lower esophageal sphincter to name but a few. Additionally, contemporary literature suggests that certain bariatric procedures may impact the motor function of the esophagus and lower esophageal sphincter postoperatively.^2^, ^3^ However, no current guidelines or specific protocol exists for a thorough evaluation of preoperative swallowing and esophageal pH testing before bariatric surgery. This oversight can significantly influence the selection of bariatric procedures. Therefore, a comprehensive preoperative assessment of the physiological functions of the esophagus should be undertaken for more effective clinical practice not only to impact weight loss in the long term but also to avoid any esophageal complications in the future. Therefore, we conducted a systematic literature search and an in‐depth review on morbid obesity, PEMD, and GERD to assess the current evidence and perspectives regarding the use of preoperative esophageal physiological testing in patients with morbid obesity undergoing bariatric surgery and its impact on choosing the most appropriate bariatric procedure.

## 2 MATERIAL AND METHODS

### 2.1 Search strategies and data collection

**FIGURE 1:**
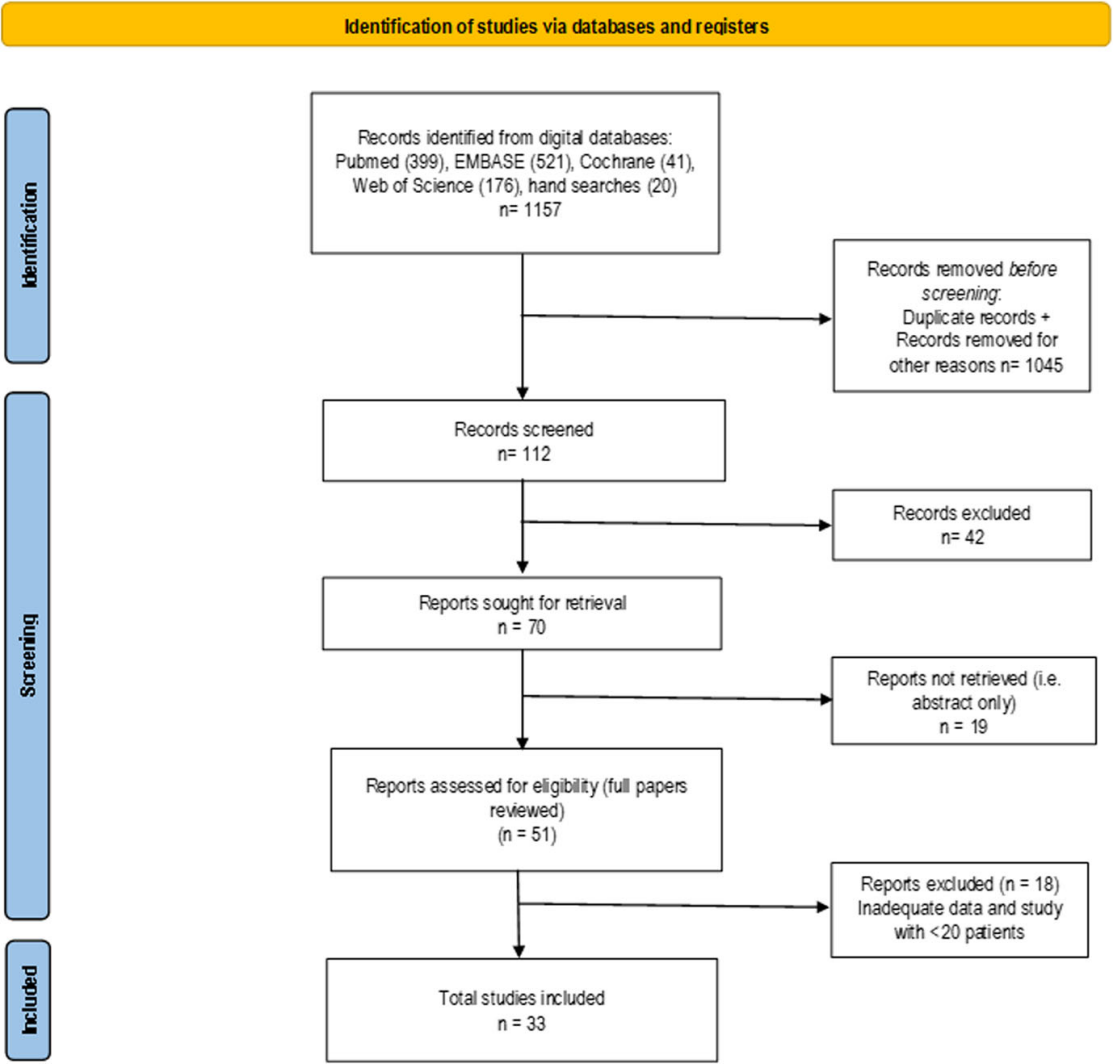
PRISMA flow chart.

Electronic databases (Medline, PubMed, EMBASE, Cochrane Register of Systematic Reviews, Science Citation Index) were searched extensively to identify studies using either conventional or high‐resolution manometry (HRM) and/or ambulatory pH study before bariatric surgery (Figure 1). The search terms were selected for each search engine to optimize and identify all published papers that met the inclusion criteria. Search strategies utilized included combinations of “laparoscopy”[MeSH Terms] OR “laparoscopy”[All Fields] OR “laparoscopic”[All Fields]), “gastric sleeve”[All Fields] OR “sleeve gastrectomy” OR “vertical sleeve gastrectomy” [All Fields] “Roux‐en‐y”[All Fields] OR “gastric bypass”[All Fields] “adjustable gastric band”[All Fields] “gastroesophageal reflux disease”[All Fields] OR “gastro‐oesophageal reflux disease”[All Fields] “weight loss surgery”[All Fields] “bariatric surgery”[All Fields] “manometry”[All Fields] “esophageal OR oesophageal function”[All Fields] “esophageal OR oesophageal motility disorder”[All Fields] “esophageal OR oesophageal motor disorder”[All Fields] “esophageal OR oesophageal dysmotility”[All Fields] AND “outcomes”[All Fields]. The reference lists of all the retrieved articles were examined for additional citations. One author (MAM) conducted a literature search and selected records that confirmed compliance with the inclusion criteria. The same author extracted data from selected studies.

### 2.2 Inclusion criteria

All studies designed to assess diagnostic and predictive test accuracy for correct discrimination of preoperative objective esophageal physiological outcome data (PEMD/GERD) for patients with morbid obesity undergoing bariatric procedures, such as laparoscopic adjustable gastric banding (LAGB), laparoscopic vertical sleeve gastrectomy (LVSG), laparoscopic Roux‐en‐Y gastric bypass (LRYGB), etc., were included. Studies performing either conventional or high‐resolution manometry (HRM) ± 24‐hour pH study were considered eligible for our analysis. Studies containing additional preoperative data in the form of patients' preoperative GER symptom assessments (structured or unstructured questionnaires) and preoperative gastroscopy ± histology were also included. No language restrictions were placed, and articles published between January 1999 and November 2023 were included in the analysis.

### 2.3 Exclusion criteria

Non‐human studies, duplicate studies, abstracts, conference articles, opinion pieces, editorial letters, case studies, reviews, and meta‐analyses were excluded from the final review. Studies with missing or inadequate manometry data and those with <20 patients were also excluded from the final review.

### 2.4 Quality assessment

We used the modified AXIS as a critical appraisal tool to assess the quality of the cross‐sectional studies^4^ (Table 1). Certain modifications of AXIS are required, as these studies were performed for diagnostic accuracy (abnormal esophageal physiology) to correctly identify patients with target conditions, that is, morbid obesity.

**TABLE 1:** Modified AXIS Table.

	Jaffin/1999^1^	Hong/2004^5^	Suter/2004^6^	Koppman/200^7^	Merrouche/2007^8^	Mejia‐Rivas/2008^9^	Valezi/2012^10^	Burgerhart/2014^11^	Cote‐Daigneault/2014^12^	Dei Genio/2014^13^	Martin‐Perez/2014^14^	Tolone/2014^15^	Mora/2016^16^	Tolone/2016^17^	Valezi/2017^3^	Coupaye/2018^18^	Schneider/2018^19^	Kristo/2019^20^	Mazzini/2019^21^	Castagneto‐Gissey/2020^22^	Greilsamer/2020^23^	Kavanagh/2020^24^	Kristo/2020^25^	Quero/2020^26^	Santonicola/2020^27^	Tolone/2020^28^	Yen/2020^29^	Chern/2021^30^	Lemme/2021^31^	Popescu/2021^32^	Soliman/2021^33^	Dalal/2022^34^	Sillcox/2023^35^
Introduction																																	
Were the aims/objectives of the study clear?	Y	Y	Y	Y	Y	Y	Y	Y	Y	Y	Y	Y	Y	Y	Y	Y	Y	Y	Y	Y	Y	Y	Y	Y	Y	Y	Y	Y	Y	Y	Y	Y	Y
Methods																																	
Was the study design appropriate for the stated aim(s)?	Y	Y	Y	Y	Y	Y	Y	Y	Y	Y	Y	Y	Y	Y	Y	Y	Y	Y	Y	Y	Y	Y	Y	Y	Y	Y	Y	Y	Y	Y	Y	Y	Y
Was the sample size justified?	N	N	N	N	N	N	Y	N	N	N	N	N	N	N	N	N	N	N	N	Y	N	N	N	N	N	N	N	N	N	N	N	N	N
Was the target/reference population clearly defined? (Is it clear who the research was about?)	Y	Y	Y	Y	Y	Y	Y	Y	Y	Y	Y	Y	Y	Y	Y	Y	Y	Y	Y	Y	Y	Y	Y	Y	Y	Y	Y	Y	Y	Y	Y	Y	Y
Was the sample frame taken from an appropriate population base so that it closely represented the target/reference population under investigation?	Y	Y	Y	Y	Y	Y	Y	Y	Y	Y	Y	Y	Y	Y	Y	Y	Y	Y	Y	Y	Y	Y	Y	Y	Y	Y	Y	Y	Y	Y	Y	Y	Y
Was the selection process likely to select subjects/participants that were representative of the target/reference population under investigation?	Y	Y	Y	Y	Y	Y	Y	Y	Y	Y	Y	Y	Y	Y	Y	Y	Y	Y	Y	Y	Y	Y	Y	Y	Y	Y	Y	Y	Y	Y	Y	Y	Y
Were the risk factor and outcome variables measured appropriate to the aims of the study?	Y	Y	Y	Y	Y	Y	Y	Y	Y	Y	Y	Y	Y	Y	Y	Y	Y	Y	Y	Y	Y	Y	Y	Y	Y	Y	Y	Y	Y	Y	Y	Y	Y
Were the risk factor and outcome variables measured correctly using instruments/measurements that had been trialed, piloted or published previously?	Y	Y	Y	Y	Y	Y	Y	Y	Y	Y	Y	Y	Y	Y	Y	Y	Y	Y	Y	Y	Y	Y	Y	Y	Y	Y	Y	Y	Y	Y	Y	Y	Y
Is it clear what was used to determine statistical significance and/or precision estimates? (eg, p values, CIs)	Y	N	Y	N	Y	Y	Y	Y	Y	Y	Y	Y	Y	Y	Y	Y	Y	Y	Y	Y	Y	N	Y	Y	Y	Y	Y	Y	N	Y	Y	Y	Y
Were the methods (including statistical methods) sufficiently described to enable them to be repeated?	Y	N	Y	N	Y	Y	Y	Y	Y	Y	N	Y	Y	Y	Y	Y	Y	Y	Y	Y	Y	N	Y	Y	Y	Y	Y	Y	N	Y	Y	Y	Y
Results																																	
Were the basic data adequately described?	Y	Y	Y	Y	Y	Y	Y	Y	Y	Y	Y	Y	Y	Y	Y	Y	Y	Y	Y	Y	Y	Y	Y	Y	Y	Y	Y	Y	N	Y	Y	Y	Y
Were the results internally consistent?	DNK	DNK	DNK	DNK	DNK	DNK	DNK	DNK	DNK	DNK	DNK	DNK	DNK	DNK	DNK	DNK	DNK	DNK	DNK	DNK	DNK	DNK	DNK	DNK	DNK	DNK	DNK	DNK	DNK	DNK	DNK	DNK	DNK
Were the results for the analyses described in the methods, presented?	Y	Y	Y	Y	Y	Y	Y	Y	Y	Y	Y	Y	Y	Y	Y	Y	Y	Y	Y	Y	Y	Y	Y	Y	Y	Y	Y	Y	Y	Y	Y	Y	Y
Discussion																																	
Were the authors' discussions and conclusions justified by the results?	Y	Y	N	Y	Y	Y	Y	Y	Y	Y	Y	Y	Y	Y	Y	Y	Y	Y	Y	Y	Y	Y	Y	Y	Y	Y	Y	Y	Y	Y	Y	Y	Y
Were the limitations of the study discussed?	N	N	N	Y	N	N	N	Y	N	Y	N	N	N	Y	N	Y	Y	Y	Y	Y	Y	N	Y	Y	Y	Y	Y	Y	Y	Y	Y	Y	Y
Other																																	
Were there any funding sources or conflicts of interest that may affect the authors' interpretation of the results?	NA	NA	NA	NA	NA	NA	N	N	N	N	N	N	N	N	N	N	N	N	N	Y	N	N	N	NA	N	NA	N	N	N	N	N	N	N
Was ethical approval (EA) or consent (C) of participants attained?	NA	C=Y EA = NA	NA	EA = Y	Y	N	C=NA EA = Y	NA	C=NA EA = Y	C=Y EA = N	N	Y	Y	Y	Y	Y	Y	Y	Y	Y	Y	C=NA EA = Y	Y	Y	Y	Y	Y	C=NA EA = Y	N	Y	Y	C=NA EA = Y	N

C = Consent, EA = Ethical Approval, DNK = Do not know, N=No, NA = Not available, Y = Yes.

### 2.5 Study design

After screening the titles and abstracts, articles that fulfilled the inclusion criteria were identified, and their full texts were reviewed (Figure 1). The data were extracted and stored in Microsoft Word Tables (Microsoft Corporation, Redmond, Washington, USA). The following data were extracted from each article: authors, year of publication, country of publication, type of bariatric surgery, type of study, number of patients, sex, age, preoperative BMI, esophageal symptoms, conventional or HRM findings, endoscopic ± histologic esophageal findings, and 24‐hour pH study findings.

### 2.6 Data extraction (outcome variables)

The primary outcome variables were the prevalence of preoperative esophageal physiological abnormalities in both esophageal manometry and the 24‐hour pH study. The secondary variables analyzed included preoperative esophageal symptoms and preoperative gastroscopy ± histologic findings.

## 3 RESULTS

We identified 33 studies^1^, ^3^, ^5^, ^6^, ^7^, ^8^, ^9^, ^10^, ^11^, ^12^, ^13^, ^14^, ^15^, ^16^, ^17^, ^18^, ^19^, ^20^, ^21^, ^22^, ^23^, ^24^, ^25^, ^26^, ^27^, ^28^, ^29^, ^30^, ^31^, ^32^, ^33^, ^34^, ^35^ (prospective = 21,^1^, ^3^, ^6^, ^8^, ^9^, ^11^, ^12^, ^14^, ^15^, ^16^, ^17^, ^18^, ^20^, ^21^, ^22^, ^26^, ^27^, ^28^, ^29^, ^30^, ^32^ retrospective = 12^5^, ^7^, ^10^, ^13^, ^19^, ^23^, ^24^, ^25^, ^31^, ^33^, ^34^, ^35^) that conducted esophageal motility/motor functions ± pH studies or both. The countries that contributed to these studies included Italy 6,^13^, ^15^, ^17^, ^22^, ^27^, ^28^ USA 6,^1^, ^5^, ^7^, ^24^, ^34^, ^35^ France 5,^8^, ^18^, ^23^, ^26^, ^33^ Brazil 4,^3^, ^10^, ^21^, ^31^ Austria 2,^20^, ^25^ Spain 2^14^, ^16^ Switzerland 2,^6^, ^19^ and one each by Australia,^30^ Canada,^12^ Mexico,^9^ the Netherlands,^11^ Romania,^32^ Tiawan,^29^ and the UK.^27^ One collaborative study was jointly conducted by authors from Italy and the UK.^27^ The largest study from Switzerland contributed 1225 patients^19^ whereas the smallest study was conducted in the USA with 20 patients only.^34^ The three most common bariatric procedures were LAGB, LVSG, and LRYGB (Tables 2, 3, and 4). Only one study^24^ used these physiological tests as the main source of clinical decision‐making for choosing bariatric procedures for their patients, and a few studies^3^, ^9^, ^10^, ^11^, ^13^, ^18^, ^22^, ^26^, ^28^, ^30^ performed postoperative testing (longitudinal studies) to assess the impact of various bariatric procedures on postoperative esophageal physiology. Excluded studies due to a lack of appropriate data included Fisher et al,^36^ Brahetto et al,^37^ Kleidi et al,^38^ Rebecchi et al,^39^ Moon et al,^40^ Ruiz de Angulo,^41^ Gemici,^42^ and Poggi^43^ and one study with <20 patients.^44^

**TABLE 2:** Conventional manometry Table.

Authors/Yr/country	Patients (M/F)	Type of bariatric surgery	Study	BMI mean±SD* or median and IQR	Age mean±SD* or median and IQR	Esophageal symptoms	Conventional manometry	LES abnormalities	Endoscopy/histology esophageal findings	24‐Hr pH
	n			Kg/m^2^	Yrs		Abnormal n (%)	n (%)	n (%)	n (%)
Jaffin et al/1999/USA^1^	111 (23/88)	LAGB	Prospective	50.7 ± 9.4*	39.9 ± 64.1*	GERD 35 (31.5%) >1 esophageal symptoms 6 (5%)†	68 (61%) Achalasia 1 (0.9%) DES 8 (7.2%) NEMD 15 (13.5) NE 16 (14.4%)	Hypotensive 39 (35.1%)	NP	NP
Hong et al/2004/USA^5^	61 (6/55)	Not mentioned	Retrospective	50.1 ± 7.2*	44.4 ± 10.3*	GERD 24 (39.3%) NCCP 1 (1.6%)‡	DES 2 (3%) NE 3 (5%) IEM 1(2%) NED 14 (23%)	Hypotensive 10 (16%) Hypertensive 11 (18%)	NP	30 (49.1%) (12 pts symptomatic)
Suter et al/2004/Switzerland^6^	345 (70/275)	Not mentioned	Prospective	44.7 (35–66.8)	38.1 (19–64)	GERD 119 (35.8%)‡	n = 332 85 (25.6%) NE 16 (4.8%) NSMD 14 (4.2%)	Hypotensive 59 (17.7%) Hypertensive 4 (1.2%)	n = 344 HH 181 (52.6%) RE 108 (31.5%)	n = 315 252 (73%) at least one parameter was abnormally elevated DMS ≥ 14.7163 (51.7%)
Koppman et al/2006/USA^7^	116 (35/81)	LAGB	Retrospective	42.9 (35–53)	48.6 (14–73)	NCCP 1 (1%)‡	NSMD 26 (23%) NE 12 (11%) DES 1 (1%) Achalasia 1 (1%)	Hypotensive 3 (3%) Hypertensive 3 (3%)	NP	NP
Merrouchie et al/2007/France^8^	100 (16/84)	LAGB/LRYGB	Prospective	45.1 (35.4–63.7)	38.4 (16–60)	Heartburn 61 (61%) Regurgitation 44 (44%) Epigastric pain 23 (23%)‡	41 (41%) decreased esophageal contraction amplitude 59 (59%) normal amplitude	Hypotensive 69 (69%) Amplitude of esophageal decreased 41 (41%)	n = 94 HH 37 (39.3%) RE 6 (6.3%) Incompetent cardia 14 (14.8%)	46 (46%) (pH < 4) 44 (44% > 50 reflux/day) DMS ≥ 14.7 49 (53.3%)
Mejia‐Rivas et al/2008/Mexico^9^	20 (4/16)	LRYGB	Prospective	48.5 ± 6.2*	38.9 ± 6.9*	GERD†	NE 2 (10%) NSMD 1 (5%)	Incompetent 4 (20%)	NP	100% abnormal DMS
Valezi et al/2012/Brazil^10^	81 (22/59)	LRYGB	Retrospective	44.9 (35–58)	44.6 (22–61)	Not mentioned	n = 37 (45.6%) abnormal Increase wave amplitude of contraction16 (43.2%) Abnormal peristalsis 3 (8.1%)	Hypotonia 7 (18.9%) Hypertonia 11 (29.8%)	NP	NP
Cote‐Daigneault et al/2014/Canada^12^	53 (16/37)	AGB	Prospective	46 ± 7.0*	43 ± 10.0*	Pyrosis 23 (43.3%) Heartburn 23 (43.3%) Regurgitation 9 (16.9%) Dysphagia 15 (28.3%) Chest pain 2(3.7%)†	Esophageal peristaltic abnormalities 27 (51%)	Hypotonia 2 (7%) Hypertonia 1 (4%) Incomplete release 4 (15%)	NP	NP
Martin‐Perez et al/2014/Spain^14^	88 (18/70)	Not mentioned	Prospective	45 ± 5.15*	41 ± 10.43*	GERD 35 (40%)†	Abnormal 49 (56.3%)	Hypotonia 40 (46%)	HH 18 (20%) RE 4 (4.5%) BE 2 (2.9%)	Positive DMS 57 (65%)
Mora et al 2016/Spain^16^	224 (55/169)	Not mentioned	Prospective	50.2 (31.7–75.6)	39.5 (18–63)	Heartburn 114 (50.9%) Regurgitation 64 (28.6%) Dysphagia 34 (15.1%) Chest pain (7.5%)‡	NE 21 (9.5%) DES 3 (1.3%) IED 7 (3.2%) NSED 5 (2.3%)	Hypotensive 47 (21.2%) Hypertensive 1 (0.4%) Incomplete relaxation 6 (2.7%)	HH 28 (12.5) RE 34 (17.3)	Abnormal pH 120 (54.2%)
Valezi et al/2017/Brazil^3^	73 (18/55)	LVSG	Prospective	41.1 (35–46)	40.2 (19–61)	Not mentioned	Increase in contraction wave amplitude 11 (31%)	Hypotonia 8 (22%) Hypertonia 17 (47%)	NP	NP
Schneider et al 2018/Switzerland^19^	1225 (352/873)	LVSG/LRYGB	Retrospective	44.4 ± 6.4*	42.2 ± 12.4*	Not mentioned	n = 610 Altered peristalsis 104 (17%)	Abnormal sphincter pathology 88 (14.4%)	n = 1190 HH 325 (27.6%) RE 229 (19.2%) Barrett's 4 (0.25%) Achalasia 1 (0.08%) Papilloma 1 (0.08%) Diverticulum 2 (0.16Z %)	NP
Mazzini et al/2019/Brazil^21^	93 (18/75)	LVSG	Prospective	41.7 ± 5*	37 ± 15.2*	GERD 50 (53.8%)†	NE 5 (5.4%)	Hypotonic 38 (40.9%) Defective 42 (45.2%) Hypertonic 13 (14%)	HH 5 (5.4%) RE 49 (52.7%)	33 (35.5%) Positive DMS
Lemme et al/2021Brazil^31^	114 (21/93)	Not mentioned	Retrospective	36 (19–64)	45.3 (40–52)	Heartburn±Regurgitation 43 (38%)‡	Abnormal 51 (45%) IEM 13 (25%) NE 10 (19%) Aperistalsis 1 (2%) Achalasia 1 (2%)	Hypotensive 16 (32%) Hypotensive+ IEM 5 (10%) Intrathoracic LES 3 (6%) Hypertensive 2 (4%)	n = 82 HH 13 (36%) EE 13 (42%) HH + EE 10 (28%)	Abnormal reflux 60 (53%) Biposition GERD 25 (42%) Supine GERD 20 (33%) Erect GERD (15 (25%)

DES = Distal or Diffuse esophageal spasm, DMS = DeMeester Score, EGJOO = Esophagogastric junction outflow obstruction, EE = Erosive esophagitis, GERD = Gastroesophageal reflux disease, HH = Hiatus hernia, IEM = Ineffective esophageal motility, RE = Reflux oesophagitis, LAGB = Laparoscopic adjustable gastric banding, LES = Lower esophageal sphincter, LRYGB = Laparoscopic Roux‐en‐Y gastric bypass, LVSG = Laparoscopic vertical sleeve gastrectomy, NCCP = Non cardiac chest pain, NE = Netcracker esophagus, NP = Not performed.

NED/NSED = Nonspecific esophageal disorder/dysmotility, NSEM = Nonspecific esophageal motility, NSMD = Nonspecific motility disorder.

* Mean±SD.

^†^ Symptoms based on standardized questionnaire such as Carlson‐Dent, Quality of life Scale for GERD (QoLS GERD), Reflux Disease questionnaire (RDQ), Gastrointestinal quality of life index (GIQLI), GERD symptoms assessment scale (GSAS), etc., although not all the studies have specified the type of questionnaire used.

^‡^ Symptoms assessment most likely during history taking.

**TABLE 3:** High resolution manometry (HRM) with 24‐hour pH studies without impedance.

Authors/Yr/country	Patients (M/F)	Type of bariatric surgery	Study	BMI mean±SD* or median and IQR	Age mean±SD* or median and IQR	Esophageal symptoms	HRM (§) or HRiM (║)	LES abnormalities	Endoscopy/ histology esophageal findings	24‐Hr pH study without impedance
	n			Kg/m^2^	Yrs		Abnormal n (%)	n (%)	n (%)	n (%)
Burgerhart et al/2014/Netherlands^11^	20 (4/16)	LVSG	Prospective	47.6 ± 6.1*	43 ± 12*	GERD 14 (70%)†	HH 5 (25%) Normal peristalsis 17 (86%) Normal DCI and IRP all patients§	20 (100%) normal LESP	NP	12 acid reflux episodes >5 min 8 (40%)
Tolone et al/2016/Italy^17^	138 (53/85)	Not mentioned	Prospective	45.8 ± 12*	36 ± 9.4*	GERD 98†	EJG type I 51 (36.9%) EGJ type II 48 (34.8%) EGJ type III (39 (28.3%)║	Not mentioned	Performed but no details	NP
Coupaye M et al/2018/France^18^	47 (1/46)	LVSG	Prospective	43.3 ± 5.7*	41.1 ± 9.4*	GERD 6 (13%)‡	HH 4 (8.5%) IEM 6 (12.7%)§	Normal	HH 3 (6.3%) Esophagitis 3 (6.3%)	DMS ≥ 14.7 16 (34%)
Castagneto‐Gissey et al/2020/Italy^22^	21 (4/15)	LVSG	Prospective	41.2 ± 0.9*	41.6 ± 2.8*	GERD score 7.9 ± 11.8 (normal)†	IRP 4.68 ± 0.9 (normal) DCI 2772.8 ± 399.9 (normal) DL 6.4 ± 0.4 (normal)§	21 (100%) normal LESP	EE 2 (10.5%)	Positive DMS 21 (100%)
Greilsamer et al/2020/Frence^23^	69 (15/54)	LVSG	Retrospective	47.5 ± 7.5	45.9 ± 9.8	Not mentioned	Hypercontractility 7 (10.1%) Hypoperistaltism 1 (1.4%)§	Hypotonic 21 (30.4%) Hypertonic 3 (4.3%)	HH 12 (17.4%) Esophagitis 12 (17.4%) Barrett's 1 (1.4%)	DMS > 14.72 21 (30.4%)
Kavanagh et al/2020/USA^24^	133 (42/91)	LVSG/LRYGB	Retrospective	46.8 (36.3–73.3)	43.6 (19–69)	Heartburn (64.6%) Regurgitation (20.3%) Dysphagia (12%) Atypical symptoms (3%)‡	n = 22 JE 2 (9%) JE + EGJOO 1 (4.5%) Achalasia 2 (9%) Others 4 (18%)§	Not mentioned	n = 51 HH 18 (36%) Esophagitis 9 (18%) Barrett's 2 (4%)	N = 12 DMS > 14.7 10 (83%)
Santonicola et al/2020/Italy & UK^27^	41 (5/36)	Not mentioned	Prospective	44.8 ± 5.4*	35.5 ± 9.8*	GERD 20 (48.8%)†	HH 10 (24.3%) EGJ type I 20 (48.8%) EGJ type II 4 (9.8%) EGJ type III (17 (41.5%)§	Not mentioned	HH 18 (43.9%) RE 11 (26.8%) Barrett's 1 (2.4%)	NP
Yen et al/2020/Taiwan^29^	57 (30/27)	LVSG	Prospective	40.5 (37.8–43.3)	35 (30–40)	GERD 25 (43.8%) Acid regurgitation 30 (53%) Heartburn 24 (42%)†	HH 19 (33%) EGJOO 9 (16%) Absent contractility 1 (2%) IEM 6 (11%)║	LESP normal	HH 22 (38.5) EE 38 (66.6%)	NP
Popescu et al/2021/Romania^32^	79 (35/44)	LVSG	Prospective	46.40 ± 6.0*	19–60	GERD 6 (7.59%) Regurgitation 6 (7.59%) Dysphagia 3 (3.8%) ?Odynophagia 2 (2.53%)†	Abnormal 37 (46.84%) EGJOO 19 (24.05%) IEM 10 (12.66%) DES 3 (3.8%) JE 3 (3.8%) Achalasia 2 (2.53%)§	Hypotonia 11 (13.93%) Hypertonia 21 (26.58%)	HH 23 (29.11%) RE 16 (20.25%)	NP
Soliman et al/2021/France^33^	160 (21/139)	LVSG	Retrospective	43.3 ± 4.5*	41.5 ± 9.2*	GERD 52 (32.5%)‡	HH 56 (35%) IEM 31 (19.4%)§	Hypotonic 46 (28.8%)	Hiatus hernia 27 (16.9%) RE 7 (4.4%)	Positive DMS 39 (24.4%)
Dalal et al/2022/USA^34^	20 (6/14)	LVSG/LRYGB	Retrospective	43.3*	58.9*	Heartburn 35% Dysphagia 25%‡	Abnormal HRM 15 (75%) Achalasia 1 (5%) EGJOO 3 (15%) DES 1 (5%) HCE 3 (15%) Peristaltic abnormality 7 (35%)§	Abnormal LES abnormality (high IRP) 4 (20%)	NP	NP

CI = Contractile integral, DCI = Distal contractile integral, DES = Distal or Diffuse esophageal spasm, DMS = DeMeester Score, EGJ = Esophagogastric junction, EGJOO = Esophagogastric junction outflow obstruction, EE = Erosive esophagitis, GERD = Gastroesophageal reflux disease, HCE = Hypercontractile esophagus, HH = Hiatus hernia, IEM = Ineffective esophageal motility, IGP = Intragastric pressure, IRP = integrated resting pressure, JE = Jackhammer esophagus,, LAGB = Laparoscopic adjustable gastric band, LES = Lower esophageal sphincter, LMGB = Laparoscopic minigastric bypass, LRYGB = Laparoscopic Roux‐en‐Y gastric bypass, LVSG = Laparoscopic vertical sleeve gastrectomy, NCCP = Non cardiac chest pain, NP = Not performed, NSED = Nonspecific esophageal disorder/dysmotility, NSEM = Nonspecific esophageal motility, NSMD = Nonspecific motility disorder, RE = Reflux oesophagitis.

* Mean±SD.

^†^ Symptoms based on standardized questionnaire such as Carlson‐Dent, Quality of life Scale for GERD (QoLS GERD), Reflux Disease questionnaire (RDQ), Gastrointestinal quality of life index (GIQLI), GERD symptoms assessment scale (GSAS), etc., although not all the studies have specified the type of questionnaire used.

^‡^ Symptoms assessment most likely during history taking.

^§^ High‐resolution manometry (HRM).

^║^ High‐resolution impedance manometry (HRiM).

**TABLE 4:** High resolution manometry (HRM) with 24‐hour pH study with impedance.

Authors/Yr/country	Patients (M/F)	Type of bariatric surgery	Study	BMI mean±SD* or median and IQR	Age mean±SD* or median and IQR	Esophageal symptoms	HRM (§) or HRiM (║)	LES abnormalities	Endoscopy/ histology esophageal findings	24‐Hr pH study with impedance
	n			kg/m^2^	Yrs		Abnormal n (%)	n (%)	n (%)	n (%)
Del Genio et al/2014/Italy^13^	25 (7/18)	LVSG	Retrospective	46.1 (38–58)	42 (22–62)	Asymptomatic†	IEM 10 (40%)║	25 (100%) normal LESP	NP	Normal DMS
Tolone et al/2014/Italy^15^	124 (72/52)	Not mentioned	Prospective	44.2 ± 7*	36 ± 14.8*	N = 102 Heartburn Regurgitation Dysphagia Chest pain†	HH 46 (37%) IEM 57 (46%)║	Not mentioned	HH 65 (52.4%) RE 18 (14.5%)	102 (82.2%) pathological acid exposure time EGJ I ↑acid exposure – not pathological EGJ II and III – ↑acid exposure ‐ pathological in all subjects
Kristo et al/2019/Austria^20^	147 (72/75)	Not mentioned	Prospective	44 (40.9–49.4)	41.6 (33.4–52.3)	Heartburn 49 (33.3) Regurgitation 27 (18.4) Dysphagia 5 (3.4)‡	50 (34%) EGJOO 27 (18.4%) JE 11 (7.5%) DES 6 (4.1%) IEM 3 (2%)║	Hypotensive 10 (6.8%) Hypertensive 21 (14.3%)	HH 52 (35.4) RE 46 (31.3%) Barrett's 7 (4.8%)	52 (35.4%)/60 (40.8%)% (True/borderline GERD)¶
Kristo et al 2020/Austria^25^	177 (72/105)	Not mentioned	Retrospective	44.6 (41.3–50.8)	42.6 (33.8–51.6)	Asymptomatic†	63 (35.6%) EGJOO 31 (17.5%) JE 14 (7.9%) DES 10 (5.6%) IEM 6 (3.4%) Fragmented Peristalsis 1 (0.6%) Achalasia 1(0.6%)║	Structurally defective 52 (29.4%) Hypotensive 10 (5.6%) Hypertensive 22 (12.4%)	HH 57 (32.2%) RE 46 (26%)	55 (31.1%)/ 78 (44.1% (True/borderline GERD)¶
Quero et al/2020/France^26^	23 (6/17)	LVSG/LRYGB	Prospective	42.4 ± 5.8*	36 ± 10*	Pyrosis/heartburn 7 (30.4%) Regurgitation 2 (8.6%)†	Normal amplitude of contraction and IGP§	LESP normal 23 (100%)	NP	1 patient pH 4 > 6%
Tolone et al/2020/Italy^28^	112 (??/??)	BIB/LAGB/LVSG/LRYGB/LMGB/LBIBP/LBPD	Prospective	42* (37–69)	39 ± 12*	Asymptomatic†	Normal values for IRP, CI, and IGP║	Normal LESP 112 (100%)	0 (0%) esophagitis	No details provided
Chern et al/2021/Australia^30^	25 (11/21)	LVSG	Prospective	43.2 ± 2.3*	45.7 ± 4.9*	GERD 20 (80%)†	EGJOO 2 (8%) DES 3 (12%) JE 1 (4%)§	Normal 17 (68%) Hypotensive 2 (8%) Hypertensive 6 (24%)	NP	Acid exposure time >4.3 13 (52%)
Sillcox et al/2023/USA^35^	291 (61/230)	LVSG	Retrospective	48.1 ± 8.7*	45.2 ± 10.9*	GERD 122 (41.9%)†	IEM 15 (5.2%) EGJOO 38 (13.1%) HCE 10 (3.4%) DES 4 (1.3%)║	Hypotensive 33 (11.3%)	NP	67 (23%) abnormal DMS

BIB = Bioenteric intragastic balloon, CI = Contractile integral, DCI = Distal contractile integral, DES = Distal or Diffuse esophageal spasm, DMS = DeMeester Score, EGJ = Esophagogastric junction, EGJOO = Esophagogastric junction outflow obstruction, EE = Erosive esophagitis, GERD = Gastroesophageal reflux disease, HCE = Hypercontractile esophagus, HH = Hiatus hernia, IEM = Ineffective esophageal motility, IGP = Intragastric pressure, IRP = integrated resting pressure, JE = Jackhammer esophagus,, LAGB = Laparoscopic adjustable gastric band, LBIBP = Laparoscopic biliointestinal bypass, LBPD = Laparoscopic biliopancreatic diversion, LES = Lower esophageal sphincter, LMGB = Laparoscopic minigastric bypass, LRYGB = Laparoscopic Roux‐en‐Y gastric bypass, LVSG = Laparoscopic vertical sleeve gastrectomy, NCCP = Non cardiac chest pain, NP = Not performed, NSED = Nonspecific esophageal disorder/dysmotility, NSEM = Nonspecific esophageal motility, NSMD = Nonspecific motility disorder, RE = Reflux oesophagitis.

* Mean±SD.

^†^ Symptoms based on standardized questionnaire such as Carlson‐Dent, Quality of life Scale for GERD (QoLS GERD), Reflux Disease questionnaire (RDQ), Gastrointestinal quality of life index (GIQLI), GERD symptoms assessment scale (GSAS), etc., although not all the studies have specified the type of questionnaire used.

^‡^ Symptoms assessment most likely during history taking.

^§^ High‐resolution manometry (HRM).

^║^ High‐resolution impedance manometry (HRiM).

^¶^ True/Borderline GERD: According to Lyons Consensus on 24‐hour pH study, the diagnosis of GERD is definite with acid exposure time (AET) > 6% whereas it remains uncertain and considered borderline if AET between 4% and 6%.

### 3.1 Conventional esophageal manometry

Fourteen studies^1^, ^3^, ^5^, ^6^, ^7^, ^8^, ^9^, ^10^, ^12^, ^14^, ^16^, ^19^, ^21^, ^31^ used conventional water‐perfused systems, in which three side holes are located at the same level as the distal extremity of the catheter, and three side holes are located 5, 10, and 15 cm away from the aforementioned holes. Several different PEMD have been diagnosed using this technique, such as distal esophageal spasm (DES), non‐specific esophageal motility disorder (NEMD), ineffective esophageal motility (IEM), nutcracker esophagus (NE), achalasia, and hypo‐ or hypertensive lower esophageal sphincter (LES). Some authors have also commented on abnormal peristalsis, aperistalsis, decreased esophageal contraction amplitude, and an abnormally increased wave amplitude of contraction. The details of the various manometric abnormalities for each study are presented in Table 2.

### 3.2 High resolution manometry

HRM was performed in 19 studies.^11^, ^13^, ^15^, ^17^, ^18^, ^20^, ^22^, ^23^, ^24^, ^25^, ^26^, ^27^, ^28^, ^29^, ^30^, ^32^, ^33^, ^34^, ^35^ It involves the use of a solid‐state manometric catheter with 36 circumferential sensors spaced 1 cm apart or a water perfused 20 channel catheter with one channel for gastric pressure, six channels at 1 cm intervals for the LES region, and 13 channels at 2 cm intervals, capable of circumferential pressure detection. Eight HRM studies also used impedance (HRiM).^13^, ^15^, ^17^, ^20^, ^25^, ^28^, ^29^, ^35^ Manometric abnormalities, including diffuse esophageal spasm (DES), achalasia, ineffective esophageal motility (IEM), jackhammer esophagus (JE), esophagogastric junction outflow obstruction (EGJOO), achalasia, hypo‐ and hypertonia of the LES, hiatal hernia (HH), etc., have been described based on Chicago Classification version 3.0 by a vast majority of studies, although one study^35^ updated their Chicago Classification to version 4.0. The details of various manometric abnormalities for each study are presented in Tables 3 and 4.

### 3.3 24‐Hour pH study with or without impedance

Twenty‐two studies undertook a preoperative 24‐hour ambulatory pH study.^5^, ^6^, ^8^, ^9^, ^11^, ^13^, ^14^, ^15^, ^16^, ^18^, ^20^, ^21^, ^22^, ^23^, ^24^, ^25^, ^26^, ^28^, ^30^, ^31^, ^33^, ^35^ Fourteen studies^5^, ^6^, ^8^, ^9^, ^11^, ^14^, ^16^, ^18^, ^21^, ^22^, ^23^, ^24^, ^31^, ^33^ used pH studies without impedance (Tables 2,3) where remaining eight studies^13^, ^15^, ^20^, ^25^, ^26^, ^28^, ^30^, ^35^ used multichannel intraluminal impedance techniques (Table 4). Some studies used the standard criteria of DeMeester Score of ≥ 14.7 as a cut‐off point differentiating between normal and abnormal acid exposure. Other studies used different criteria for designating the pH study as abnormal, such as total time with pH < 4 min, total number of reflux episodes, number of episodes >5 min, and bipositional GERD. The important pH findings for each study are described in Tables 2, 3, 4.

### 3.4 Gastroscopy ± histology

Twenty studies^6^, ^8^, ^14^, ^15^, ^16^, ^17^, ^18^, ^19^, ^20^, ^21^, ^22^, ^23^, ^24^, ^25^, ^27^, ^28^, ^29^, ^31^, ^32^, ^33^ performed preoperative gastroscopy ± histology on their patients. The incidence of hiatal hernia (HH) varied from 5.4% to 52.6%,^6^, ^21^ and that of reflux esophagitis (RE) from 4.4% to 42%.^31^, ^33^ Other pathologies include Barrett's esophagitis (BE), achalasia, esophageal papilloma, and esophageal diverticulum. The details of the various endoscopic abnormalities and histologic findings for each study are described in Tables 2, 3, 4.

### 3.5 GERD symptoms

Eighteen studies^1^, ^9^, ^11^, ^12^, ^13^, ^14^, ^15^, ^17^, ^21^, ^22^, ^25^, ^26^, ^27^, ^28^, ^29^, ^30^, ^32^, ^35^ used validated GERD symptoms questionnaires such as Carlson‐Dent Questionnaire,^9^ Reflux Disease Questionnaire,^11^ Quality of Life Scale for Gastroesophageal Reflux Disease,^21^ GERD Health‐Related Quality of Life,^22^ etc. Eleven studies^5^, ^6^, ^7^, ^8^, ^16^, ^18^, ^20^, ^24^, ^31^, ^33^, ^34^ undertook subjective evaluation of preoperative esophageal symptoms and identified patients with preoperative GERD using terms such as heartburn, dysphagia, odynophagia, regurgitation, chest pain, pyrosis, GERD, etc., and gave these symptoms numeric values (0–4) whereas four studies failed to provide any symptoms evaluation^3^, ^10^, ^19^, ^23^ (Tables 2, 3, and 4).

## 4 DISCUSSION

Several studies have investigated the occurrence and relevance of preoperative PEMD in patients with morbid obesity before undergoing bariatric surgery (Tables 2, 3, and 4). This is because of the growing body of evidence suggesting that these patients have a higher prevalence of PEMD. However, the underlying mechanisms remain unclear. Nevertheless, there are speculations regarding the role of a fatty diet, which may have an inhibitory effect on the LES or increase the frequency of distension‐induced transient lower esophageal sphincter relaxations (TLESRs).^45^ Whether this effect is mediated by the secretion of hormones, such as secretin and cholecystokinin is speculative.^46^ Two further studies on the role of high‐fat and high‐calorie content on postprandial GERD have shown that the percentage of time at pH < 4 after a high‐fat, high‐calorie meal was almost double of that after a low‐fat, low‐calorie meal.^47^, ^48^ Whether the direct and damaging effects of stomach acid also play a role in the causation of esophageal motor dysfunction has been greatly debated. Some of the critical factors in the pathogenesis of GERD are (a) the role of LES tone and (b) the frequency and duration of TLESRs, which seem to be more common in individuals with obesity. Quiroga et al^49^ have demonstrated that, patients who are non‐obese with GERD, when compared with patients with obesity and GERD, had a greater incidence of esophageal dysmotility, including impaired complete bolus transit. Fornari et al^50^ furthermore have shown that patients with obesity differed from patients who are non‐obese in terms of esophageal motility and reflux regardless of the presence of GERD. Patients with obesity show stronger peristalsis and increased acid exposure in the esophagus. Pandolfino et al^51^ analyzed 285 patients with obesity to determine the relationship between obesity and morphology of the esophagogastric junction (EGJ) pressure segment using HRM. They found a direct linear relationship between BMI, waist circumference, intragastric pressure (IGP), and the gastroesophageal pressure gradient (GEPG). The authors concluded that patients with obesity have a higher risk of EGJ disruption, leading to HH and augmented GEPG, providing a perfect condition for reflux. The increased incidence of a dual high‐pressure zone may also explain the increased number of TLESRs and reflux events using postprandial impedance‐pH monitoring in patients with obesity.^52^ Tolone et al^15^ similarly reported a prospective study of preoperative HRM in 124 patients with morbid obesity. The mean GEPG was significantly higher in patients with obesity than in volunteers who were not obese. Furthermore, the separation between the LES and crural diaphragm increased compared to volunteers who were not obese. The authors also reported a higher prevalence of IEM in individuals with morbid obesity, especially in those who also had an abnormal gastroscopy and/or an abnormal 24‐hour impedance‐pH study. A larger study with a slightly larger cohort of 138 patients by the same authors^17^ confirmed that the greater the separation between the LES and the crural diaphragm, the greater the risk of GERD. They further demonstrated that, compared to individuals who were non‐obese, patients with obesity have a higher risk of HH, lower LES resting pressure, lower distal contractile integral, and increased incidence of IEM.

The very first large‐scale study published by Jaffin et al^1^ utilizing water‐perfused esophageal manometry equipment in 111 patients with morbid obesity found that 61% of their subjects had at least one abnormality on esophageal manometry (Table 2). Furthermore, 39 (35.1%) patients had hypotensive LES, which increased the risk of GERD. The striking finding of this study was that 68 patients (58.8%) with PEMD were asymptomatic. The authors did not perform either a preoperative gastroscopy or 24‐hour ambulatory pH study to consolidate their manometry findings. Following this initial study, several other prospective and retrospective studies published their preoperative esophageal manometry data along with a 24‐hour ambulatory pH study for bariatric patients (Tables 2,3, and 4). Hong et al^5^ confirmed a high incidence of PEMD (54%) in a cohort of 61 patients with morbid obesity. Suter et al^6^ undertook conventional manometry in 332 patients, which revealed esophageal motility abnormalities in 85 patients (25.6%), including low LESP in 17.7%, NE in 4.8%, and NSMD in 4.2%. A number of other studies utilizing conventional manometry techniques have similarly demonstrated a high incidence of PEMD in patients with morbid obesity ranging from 17% to 51%: Koppman 23%,^7^ Merrouche 41%,^8^ Valezi 45.6%,^10^ Cote Daigneault 51%,^12^ Mora 33.4%,^16^ Schneider 17%,^19^ and Lemme 45%^31^ and varying incidence of hypotensive LES (3%–69%).^7^, ^8^ The largest retrospective study involving 1225 patients by Schneider et al^19^ however has shown a low incidence of PEMD (17%); however, the exact details of various esophageal motor dysfunctions are lacking.

The introduction of high‐resolution manometry (HMR) in 1990 provided more insight into the physiological motor dysfunction of the esophagus. It has several advantages over conventional manometry and includes (1) simultaneous visualization of the contractility of the entire esophagus in a uniform standardized format, (2) systematic application of standardized objective metrics of peristaltic and sphincter function for interpretation, and (3) easy recognition of topographic patterns of contractility with greater reproducibility.^53^ Therefore, several authors have undertaken preoperative HRM assessments of patients with morbid obesity, providing a more accurate account of the state of affairs of PEMD and LES topography. Two of the largest studies, one from Austria (n = 177 patients)^25^ and the other from the USA (n = 291 patients),^35^ confirmed a high incidence of PEMD (35.6% and 22.7%, respectively; Table 4). Furthermore, structurally defective LES, whether hypo‐ or hypertensive, was found in a substantial number of patients (11.3%–29.4%).^25^, ^35^ In Kristo's study,^25^ all their 177 patients were asymptomatic. Analysis of their data revealed GERD in 55 (31.1%) patients and borderline GERD in 78 (44.1%) patients (Table 4), which was mediated by structurally defective LES in 64 (36.2%) patients and highlighted by acidic and nonacidic reflux episodes on 24‐hour pH impedance manometry. PEMD detected using HRM included EGJOO in 31 (17.5%), JE in 14 (7.9%), DES in 10 (5.6%), IEM in 6 (3.4%), fragmented peristalsis in 1 (0.6%), and type III achalasia in 1 (0.6%) patient. Concomitant EGJOO was noted in 9 (64.3%) patients with JE and 6 (60%) patients with DES. Gastroscopy revealed HH in 57 patients (32.2%) and esophagitis in 46 patients (26%). Patients with GERD had a shorter total LES length and shorter intra‐abdominal LES fraction. This study demonstrated that even in completely asymptomatic patients, there is a high risk of both GERD and PEMD. In the Sillcox study,^35^ 67 patients (23%) had an abnormal DeMeester Score. EGJOO (13.1%) was the most prominent disorder. Although both the conventional and HRM have shown significant populations of patients with morbid obesity suffer from some sort of PEMD, comparison between these two techniques is challenging due to revisions in esophageal manometry nomenclature, for example, nutcracker esophagus (NE), which was considered hypertensive peristalsis, has been omitted from the recent Chicago Classification due to its unclear clinical significance. However, a new terminology, jackhammer esophagus (JE), which refers to a hypercontractile esophagus, was introduced in the Chicago Classification 3.0 and its definition remains the same for version 4.0.^53^ This is defined as a hypercontractile esophagus with at least one contraction with distal contractile integral (DCI) using HRM must be >8000 mm Hg.s.cm and ≥20% hypercontractile swallows and is usually associated with esophageal symptoms such as dysphagia and chest pain.

Most studies have analyzed LES abnormalities using both conventional manometry and HRM (Table 2, 3, 4). The most common abnormality detected was hypotensive (or hypotonic) LES, the incidence of which varied from 3% to 69% using conventional system^7^, ^8^ and from 5.6% to 28.8% using HRM.^25^, ^33^ However, several HRM studies^11^, ^13^, ^18^, ^22^, ^28^, ^29^ have reported completely normal LES pressure (LESP) in all their patients with morbid obesity. Hypotensive LES along with HH and TLESR most likely leads to LES staying open for a prolonged period of time, resulting in severe reflux esophagitis due to greater acid exposure and reduced acid clearance.

Even though preoperative esophageal manometry may detect or raise suspicion for PEMD, the addition of other preoperative complementary investigations such as gastroscopy ± histology and 24‐hour pH monitoring may provide an additional layer of protection for choosing the most appropriate bariatric procedure, as these investigations provide an added objective assessment of the anatomy and physiology of the esophagus. Therefore, several studies (Table 2, 3) used a combination of gastroscopy +/− pH study to further investigate their patients before offering them a bariatric procedure. Suter et al^6^ analyzed data from 345 patients undergoing bariatric procedures and found that 38.5% of patients reported reflux symptoms. Endoscopy revealed HH in 181 (52.6%) patients and RE in 108 (31.4%) patients. Furthermore, they undertook a 24‐hour pH study in 315 patients, which revealed that 252 (73%) patients had at least one parameter that was abnormally elevated. The most common abnormal finding was an increased total number of reflux episodes in 61.8% of patients. Another important finding of this study was that patients with increased exposure to acid in the upright position had a larger waist‐to‐height ratio (WHR) compared to those with normal exposure. Patients with GER symptoms were significantly more likely to have elevated DeMeester scores than their asymptomatic counterparts. The authors noted that almost half of their patients who did not report GER symptoms had an abnormal 24‐hour pH study confirming a higher prevalence of GERD, even in asymptomatic patients with morbid obesity. Lastly, the authors confirmed the negative impact of HH which significantly increases the risk of an abnormal pH study. This is because an HH usually displaces the LES from its normal position, altering the anatomic barrier to reflux in some patients. Furthermore, it acts as an acid reservoir, which, along with hypotonic LES, leads to a retrograde flow of acid into the esophagus. Additionally, HH leads to prolonged acid contact with esophageal mucosa and delayed clearance, leading to esophagitis and its consequences. A number of studies^5^, ^6^, ^8^, ^9^, ^11^, ^13^, ^14^, ^15^, ^16^, ^18^, ^20^, ^21^, ^22^, ^23^, ^24^, ^25^, ^26^, ^30^, ^31^, ^33^, ^35^ have also undertaken 24‐hour pH studies confirming a high incidence of GERD (35.5% to 100%).^9^, ^21^ Similarly, gastroscopy has also shown variable prevalence of HH (5.4%–52.6%)^6^, ^21^ and RE (4.5%–52.7%)^14^, ^21^ (Table 2, 3, 4). Ayazi et al^54^ have provided a number of important observations in their 1659 patients who underwent manometry and pH studies: (1) for every unit increase in BMI, the percent total time pH < 4 increased by 0.35% (95% CI, 0.24–0.46); 13% of the variation in esophageal acid exposure may be attributable to variation in BMI; (2) subjects who are heavier have a greater frequency of a mechanically defective LES. When compared to patients with normal weight, patients with obesity are more than twice as likely to have a defective LES, and (3) the likelihood of a defective LES is two‐fold higher with HH than without HH.

Two recent large retrospective studies, one from France^33^ and the other from the USA^35^ analyzed data to ascertain whether preoperative esophageal physiological testing predicts postoperative GER symptoms after LVSG. Soliman et al^33^ analyzed 160 patients who underwent a preoperative 24‐hour pH study and HRM. According to this study, predictive factors of postoperative symptoms are (a) preoperative symptoms and (b) a positive preoperative 24‐hr pH study in asymptomatic patients. This study also demonstrated that one‐third of their asymptomatic patients developed de novo GER symptoms, especially those with preoperative silent GERD after LVSG. Surprisingly, no manometric factors were correlated with postoperative GERD symptoms. Similarly, Sillcox et al^35^ also determined that although manometric abnormalities did not predict postoperative GER symptoms, the GERD burden did. The higher the preoperative DeMeester score was in their patients, the higher the risk of GERD. They concluded that a preoperative pH study may guide operative decision‐making and lead to better counseling of patients at risk for GERD after LVSG.

The only publication to date addressing the issue of developing a preoperative protocol in a population with obesity ± GERD to have a significant impact on the selection of bariatric procedures was published by Kavanagh et al^24^ from Iowa, USA. These authors devised a simple linear process for patients who are obese with subjective GER symptoms. During the initial consultation, the patient requested their choice of bariatric surgical procedure, that is, LVSG or LRYGB. They then underwent objective protocol testing before being evaluated for the second time, when the final consideration regarding the most optimal bariatric procedure was discussed by the treating surgeon. All the patients underwent gastroscopy and esophagography. If HH, significant reflux, or esophageal dysmotility was discovered on esophagram or if Los Angeles (LA) grade C or D esophagitis or Barrett's were discovered on gastroscopy, LRYGB was recommended. However, if a patient desired a LVSG with normal gastroscopy and esophagram but with mild GER symptoms and was not proton pump‐dependent, the authors felt that it was permissible for them to undergo LVSG. However, patients with normal gastroscopy and esophagram but classic moderate to severe GER symptoms or PPIs dependent were offered HRM and 24‐hour pH testing for further evaluation. If these patients demonstrated an abnormal DeMeester score on 24‐hour pH monitoring or dysmotility on HRM, LRYGB surgery was recommended. The authors believed that their simple algorithm objectively examined the foregut anatomy and physiology of patients with morbid obesity in detail, further impacting the final bariatric surgical choice in one in four patients (25%).

### 4.1 Strengths and limitations

This review provides real‐life preoperative data on the esophageal physiology in patients with morbid obesity. This demonstrates a high prevalence of PEMD and GERD in these patients, which may be aggravated by choosing an incorrect bariatric procedure and may even result in serious long‐term consequences, such as progression to BE, EE, and esophageal adenocarcinoma. The limitations of this review are: (a) the analysis is based on retrospective and prospective studies with unknown and unnoticed biases, unrecognized confounders, and missing data; (b) the risk of false association between the variables of interest and the outcome, even in the absence of a true association, especially when the studies lack sufficient power to account for type I or type II errors; and (c) most of these studies are classified as having low quality based on the AXIS appraisal tool. Furthermore, as most of the included studies were cross‐sectional, they could not analyze behavior over an extended period of time; therefore, it is difficult to establish a cause‐and‐effect relationship. The strength of cross‐sectional studies is that they can provide valuable and rapid analysis for particular variables (PEMD/GERD) in certain diseases/conditions (morbid obesity). This enables patients to quickly receive informed guidance and counseling regarding their preferred choice of a bariatric procedure.

### 4.2 Conclusions

The preoperative prevalence of PEMD and GERD is quite significant in patients with morbid obesity, and it is vital that a detailed preoperative review of patients' symptoms should be undertaken to reveal underlying esophageal motor dysfunctions and GER symptoms. This should then initiate a defined algorithm to undertake comprehensive objective esophageal anatomical and physiological testing. However, it is evident from these real‐life data that a substantial number of patients with morbid obesity may be asymptomatic. This is because of autonomic and sensory ganglion susceptibility due to the consequences of obesity, such as increased immune cell entry, causing damage and dysregulation of sensory pathways.^55^ Therefore, the question remains as to whether every patient with morbid obesity should be subjected to physiological testing coupled with gastroscopy to ascertain the presence of esophageal abnormalities? Would these investigations prompt surgeons to select the most appropriate bariatric procedure not just for weight reduction but also for the prevention of GERD and deterioration in esophageal motor dysfunction in the future? Therefore, a recent Lyon Consensus statement encourages physiological testing in patients with morbid obesity.^56^ According to the American Society for Metabolic and Bariatric Surgery^57^ in 2021, 262,893 bariatric surgical procedures were performed, of which 58% (152,866) were LVSG, a restrictive procedure that is associated with a higher incidence of GERD. In two recent meta‐analyses,^58^, ^59^ LVSG was associated with the development and worsening of GERD symptoms compared with LRYGB at 5 years postoperatively, leading to either introduction/increased pharmacological requirement or further surgical treatment.^58^, ^59^ It is therefore evident that without any appropriate guidelines, the use of LVSG will continue to increase, leading to an enormous health burden in terms of GERD in the future. Therefore, we feel that it is incumbent upon the surgical community to devise a guideline through a consensus of various bariatric societies worldwide, which could provide a meaningful algorithm for the selection of the best bariatric procedures for each patient based on various preoperative anatomical and physiological tests. The goal of any bariatric procedure simply should not just be weight reduction, but the prevention of both short‐ and long‐term sequelae of that particular bariatric procedure, which includes (a) GERD and its future consequences, such as BE, EE, and esophageal adenocarcinoma, and (b) progression of any esophageal motor dysfunction, which may also contribute to the development of GERD. It is expected that HRIM will advance our understanding of postoperative dynamic changes in esophageal motor function and their impact on the EGJ complex based on the anatomical and physiological changes associated with a particular bariatric procedure, which in the future may determine the type of bariatric procedure offered to the patient.

## ETHICAL COMPLIANCE

This paper is a systematic review and does not contain any human or animal participants.

## CONFLICT OF INTEREST STATEMENT

None.

## Data Availability

Research data involving various publications are available from numerous electronic databases (Medline, PubMed, EMBASE, Cochrane Register of Systematic Reviews, Science Citation Index, and journals websites.
